# *In Situ* Representation of Soil/Sediment Conductivity Using Electrochemical Impedance Spectroscopy

**DOI:** 10.3390/s16050625

**Published:** 2016-04-30

**Authors:** Xiaojing Li, Xin Wang, Qian Zhao, Yueyong Zhang, Qixing Zhou

**Affiliations:** 1 Agro-Environmental Protection Institute, Ministry of Agriculture, Tianjin 300191, China; lixiaojing@caas.cn; 2 MOE Key Laboratory of Pollution Processes and Environmental Criteria, Tianjin Key Laboratory of Environmental Remediation and Pollution Control, College of Environmental Science and Engineering, Nankai University, Tianjin 300071, China; zqtalent@163.com (Q.Z.); zyynku@126.com (Y.Z.)

**Keywords:** soil microbial fuel cells, soil electrical conductivity, electrochemical impedance spectroscopy, soil resistivity, resistance measurement apparatus

## Abstract

The electrical conductivity (EC) of soil is generally measured after soil extraction, so this method cannot represent the *in situ* EC of soil (e.g., EC of soils with different moisture contents) and therefore lacks comparability in some cases. Using a resistance measurement apparatus converted from a configuration of soil microbial fuel cell, the *in situ* soil EC was evaluated according to the Ohmic resistance (*R*_s_) measured using electrochemical impedance spectroscopy. The EC of soils with moisture content from 9.1% to 37.5% was calculated according to *R*_s_. A significant positive correlation (*R*^2^ = 0.896, *p* < 0.01) between the soil EC and the moisture content was observed, which demonstrated the feasibility of the approach. This new method can not only represent the actual soil EC, but also does not need any pretreatment. Thus it may be used widely in the measurement of the EC for soils and sediments.

## 1. Introduction

The soil electrical conductivity (EC), associated with soil salinity [1], is measured as one of main soil characteristics in agricultural production and environmental protection. It is influenced by soil moisture content, salts, and amount and type of clays and other factors [2]. Generally, the soil EC is determined by measuring the EC of extracted soil samples [1,3]. In this case, the ratios of soil to water must be ascertained, including 1:2.5, 1:5 and 1:10 [2,3,4,5]. The ECs obtained for different soil to water ratios indicate poor comparability. Although the ratios are equivalent, the comparability is doubtful due to the differences in the amount and type of ions, the time of extraction or the water temperature [6,7]. 

The use of electrochemical impedance spectroscopy (EIS) has been employed frequently in the evaluation of the electrochemical performance of microbial fuel cells (MFCs), especially the measurement of cell internal resistance [3,8,9]. The electrochemical properties related to resistances, including a charge transfer resistance (*R*_ct_), a solution resistance (*R*_s_, soil Ohmic resistance in this study) and a capacitance (*C*), are determined by simulating the measured impedance data according to an equivalent circuit [10]. In this study, a configuration of soil MFC (addressed as resistance measurement apparatus in this study) was designed to obtain the soil resistance by measuring electrochemical impedance. The *in situ* soil EC was simply determined from the slope of resistance-distance plot in a resistance measurement apparatus. So far as we know, there’s no other literature describing how to measure soil EC using EIS. This method is new and does not need any pretreatment compared to the conventional method. Since no dehydration process is needed before the measurement, it is possible to obtain soil EC for different moisture contents.

Electrochemical technologies, for instance MFC and the electrokinetic technique, are promising remediation approaches for soil/sediment contamination [11,12]. The performance of remediation is mainly determined by the system resistance [5,13]. Thus the representation of contaminated soil/sediment EC is an important aspect in electrochemical remediation. Generally, the soil/sediment EC is evaluated by analyzing their extraction solutions, which does not represent the real-time conductivity of the soil/sediment. In this study, an *in situ* representation method of EC was devised using the EIS that can continuously monitor the soil/sediment EC, which is a very important index to evaluate the soil characteristics for plant growth and soil organisms.

## 2. Materials and Methods

### 2.1. Apparatus Configuration 

**Figure 1:**
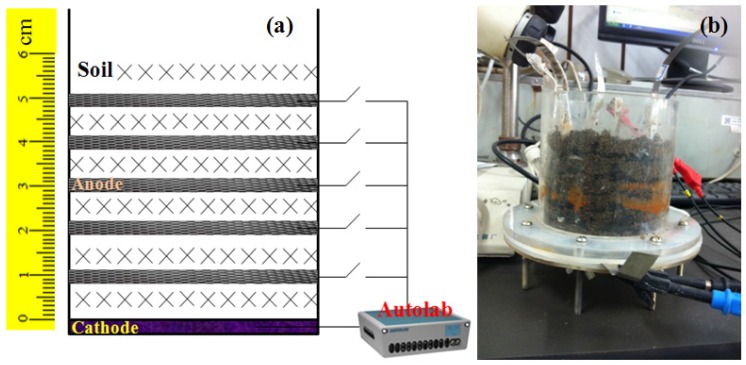
Schematic (**a**) and photograph (**b**) of the resistance measurement design.

A resistance measurement apparatus (RMA) was assembled with five layers of carbon mesh anodes and an activated carbon air-cathode (Figure 1). The soil RMA was column-type, with 10 cm of diameter and 10 cm of height. The carbon meshes (diameter of 10 cm, Jilin Carbon Factory, Jilin, China) were soaked in acetone overnight prior to use [14]. The air-cathode (diameter of 10 cm) was produced by a rolling-press method and consisted of a 60 mesh of stainless steel mesh (type 304, Detiannuo Commercial Trade Co. Ltd., Tianjin, China) with a catalyst layer (CL) rolled on the soil facing side and a gas diffusion layer (GDL) rolled on the air facing side [10]. The GDL was made of carbon black (Jinqiushi Chemical Co. Ltd., Tianjin, China) and PTFE emulsion (60 wt%, Hesen, Shanghai, China) with a mass ratio of 3:7, followed by heating at 340 °C for 20 min. The CL was made of activated carbon (Xinsen Carbon Co. Ltd., Fujian, China) and PTFE emulsion with a mass ratio of 6:1. The air-cathodes sheets were dried at room temperature for least 24 h before installed in RMA. The air-cathode was supported by a porous Plexiglas plate (0.5 cm of pore diameter and 1 cm of pore spacing between two pores) at the bottom of the RMA. Anodes were parallelly inserted in soil with distance of 1, 2, 3, 4 and 5 cm from the air-cathode (Figure 1). A titanium sheet (1 mm of thickness and 1 cm of wide) was used as the wire.

### 2.2. Tested Soil and Electrochemical Measurements

The soil was collected in a suburb of Dagang district of Tianjin, China. After being air-dried, the soil was passed through a 2 mm sieve. Next 600 g of dried soil was mixed with different amounts of distilled water to obtain test soil swith certain water contents. Since the saturated water content of the original soil is 37.5%, soils with different moisture contents of 9.1%, 16.7%, 23.1%, 28.6%, 33.3% and 37.5% were prepared by addition of distilled water (electrical conductivity of 28 μS·cm^−1^). The homogeneous test soil was divided into six parts and each part was filled in between two electrodes (anode or cathode). Each treatment had a duplicate. EIS was performed at a frequency range from 100 kHz to 10 mHz with a amplitude of 10 mV using a potentiostat (Autolab PGSTAT 302N, Metrohm, Herisau, Switzerland) at the open circuit potential. The carbon mesh anode was the working electrode, and the air-cathode was used as the counter and reference electrode (two electrodes system). Nyquist plots were used to interpret the spectra and simulated using a software program (ZsimpWin 3.10, Bruno Yeum, EChem Software, Ann Arbor, MI, USA). 

### 2.3. Characteristics Analysis

The main properties of the soil are summarized in Table 1. The soil density (*ρb*) was measured from soil dry weight/isometric water weight, and the porosity was calculated as (1-*ρb*/2.65) × 100% [15]. The pH was determined in a mixture with distilled water to soil mixture of 5:1 (*v*/*w*) [5]. The soluble salt content was extracted as described in a previous report [16]. In brief, 10 g of soil was mixed with 50 mL of CO_2_-free water and shaken 3 min. 20 mL of supernatant was evaporated at 105 °C and then H_2_O_2_ (10% of mass fraction) was used to remove organic matter. In order to make a comparison with EC measured using the conventional method, ECs of soil were evaluated with soil to distilled water ratios (*v*/*w*) of 1:2.5, 1:5 and 1:10 using a EC meter (SFENGCI, DDS-11A, Shanghai, China) [3,4]. The soil particle size distribution was obtained by using Mastersizer2000particle size analyzer (Malvern Instruments Ltd., Malvern, UK). 

### 2.4. Calculation

The soil Ohmic resistance (*R*_s_) is the mean resistance of two duplicates by using the RMA. The definition of *R*_s_ is expressed as follows:

(1) $$R_{S}=\;\rho \;\times \;\frac{L}{S}$$

where *ρ* is the soil resistivity (Ω·cm), *L* is the length of a sample (in this study, it represents the distance from anode to cathode, cm) and *S* is the cross sectional area (geometric parameter of a reactor, cm^2^).

The soil EC (*κ*, mS·cm^−1^) is the reciprocal of the soil resistivity, and can be calculated as follows:

(2) $$\kappa =\;\frac{1}{\rho }$$

## 3. Results

### 3.1. Nyquist Plots Represent R_s_

**Figure 2:**
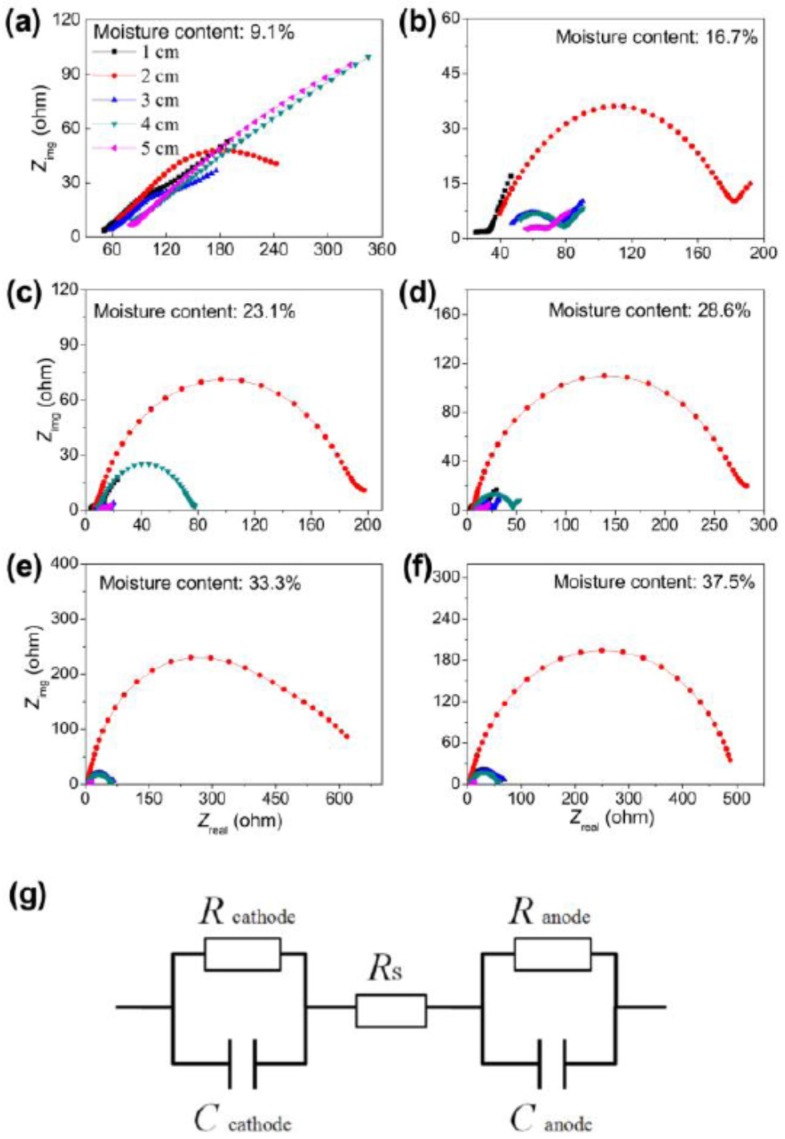
Nyquist plots (**a**–**f**) of soils of different moisture contents at open circuit potential and the equivalent circuit (**g**) for simulating electrochemical impedance spectroscopy.

The soils with different moisture contents were filled into the RMA and were stabilized for at least 1 h prior to measurement. The Nyquist plots simulated according to an equivalent circuit are shown in Figure 2 and Figure S1. Fitting results showed that the Ohmic resistance (*R*_s_) of the soil decreased with the soil moisture content. A sharp *R*_s_ decrease was observed when the moisture content of the soil increased from 16.7% to 23.1%, for example from 21.4 ± 1.9 to 3.8 ± 0.1 Ω for 1 cm of distance. It was expected that the *R*_s_ increased with the distance from the air-cathode (Table 2 and Table S1). For all RMAs, the charge transfer resistance (*R*_ct_) at the distance of 2 cm between anode and cathode showed a larger value than any other. The *R*_ct_ denotes the electron transfer capacity and thus exerted no effect on the inherent Ohmic resistance of RMA.

### 3.2. Resistivity and EC

**Figure 3:**
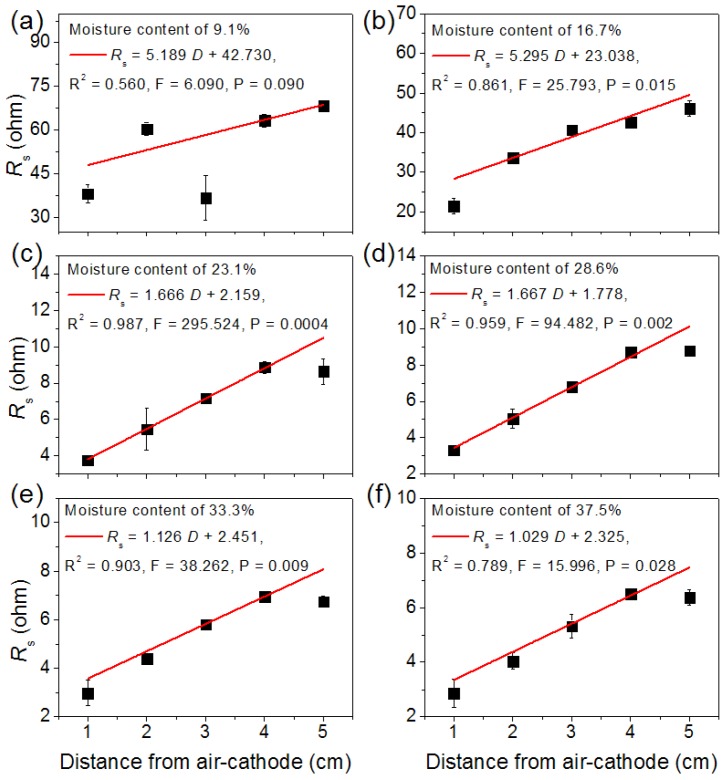
Linear fitting between *R*_s_ and distance from air-cathode at different moisture contents of soil samples: (**a**) moisture content of 9.1%; (**b**) moisture content of 16.7%; (**c**) moisture content of 23.1%; (**d**) moisture content of 28.6%; (**e**) moisture content of 33.3%; (**f**) moisture content of 37.5%.

The Ohmic resistance (*R*_s_) is theoretically proportional to the distance (*D*) between anode and cathode. According to Equation (1), the soil resistivity (*ρ*) can be calculated from the slope of the *R*_s_-*D* profile. As shown in Figure 3, the relationship between *R*_s_ and *D* for different soil moisture contents exhibited a good linear correspondence except for the soil with the lowest moisture content (9.1%). Because this sample did not contain enough water, poor contact between electrode and soil resulted in the relatively insignificant linear relationship. It was likely that this method is unsuitable for soils with <9.1% of water content. However, it was utterly applicable for sediment, which held enough water. The soil resistivity was calculated as *ρ* = slope × *S*, where *S* was the cross sectional area of the reactor (78.5 cm^2^). Therefore, the soil EC was calculated according to Equation (2) (Table 3). With the increase of water contents, the soil resistivity decreased while the soil EC increased, indicating that this method provided a good representation of soil conductivity. 

### 3.3. Carbon Mesh Layers Inside Soil

**Figure 4:**
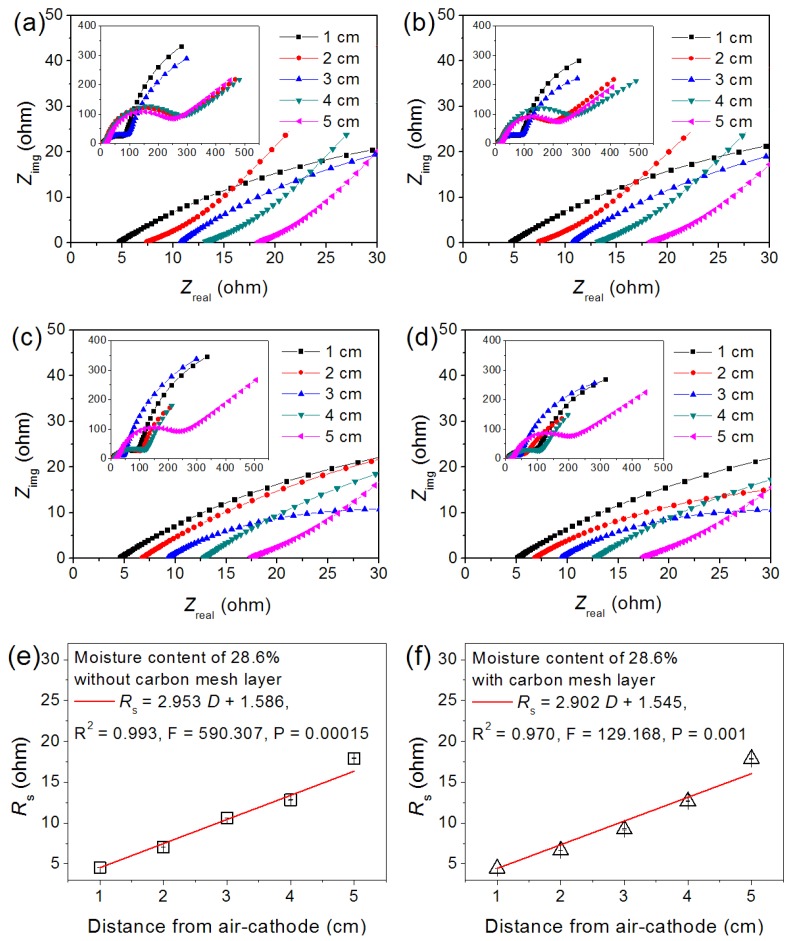
Nyquist plots measured with multiple (**a**,**b**; duplicated values) and single (**c**,**d**; duplicated values) layer of carbon mesh. Linear fittings of multiple (**e**) and single layer (**f**) of carbon mesh between mean values of *R*_s_ and electrode distance (28.6% of soil moisture content).

In order to evaluate the effect of multiple layers of carbon mesh on the soil EC measurements, an anode/soil/cathode assembly (only one layer of carbon mesh in soil) was tested at a soil moisture content of 28.6% (the median value used as a typical sample). It was confirmed that the addition of carbon mesh did not result in a significant increase in Ohmic resistance. For example, the *R*_s_ values with electrode spacing of 4 cm were 12.7 ± 0.04 and 12.83 ± 0.02 Ω for single and multiple anode measurements. As shown in Figure 4 and Table 4, the soil ECs measured using single and multiple carbon mesh anodes were 4.312 and 4.387 mS·cm^−1^, with no significant difference between them, showing that the possible resistance caused by multiple carbon mesh layers was ignorable. Notably, the soil EC here was smaller than the previous result (7.638 mS·cm^−1^) due to the fact this measurement was conducted in winter (~15 °C) while the previous was in summer (~35 °C), showing that the temperature had an important effect on the soil EC. However, compared to the multiple anodes system, the use of a single anode required reconstructing this system many more times than multiple anodes to obtain *R*_s_ values at different electrode spacings. That was to say, the RMA with multiple carbon mesh layers was a simple and efficient representation for the soil EC. 

## 4. Discussion

**Figure 5:**
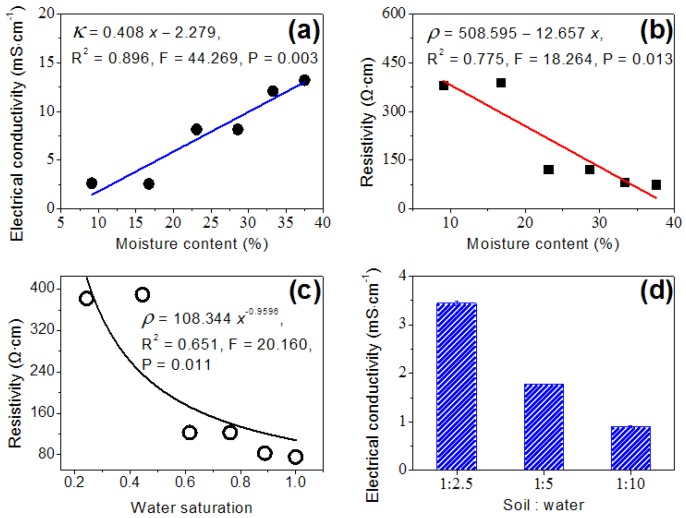
Electrical conductivity (**a**); resistivity (**b**,**c**) as the function of soil moisture contents and water saturation (water content/saturated water content); and the soil electrical conductivity (**d**) measured by using conductivity meter at different ratios of soil to water.

The soil EC, derived from the soil resistivity, was obtained by EIS in this study. According to a previous report [3], the soil EC < 1 cm to the anode was enhanced from 8.91 to 13.4 mS·cm^−1^ when the soil moisture content increased from 23% to 33%. A similar trend was also found in this study. In addition, there was a significant linear correlation between the soil ECs and the moisture contents (*R*^2^ = 0.896, *p* = 0.003, Figure 5a). Likewise, a significant linear correlation between the soil resistivities and the moisture contents (*R*^2^ = 0.775, *p* = 0.013, Figure 5b) was also observed. The trend of resistivity with the water saturation accorded with Archie formula, and the coefficient of determination and the exponent of water saturation were 0.651 and −0.9596 (F = 0.011, Figure 5c). 

The soil EC, measured according to the conventional method [3,5], is a cursory response for the water soluble salt. The principle is that the water soluble salt content of soil is positively correlated with the soil EC under certain conditions. Thus, different soil moisture contents resulted in obviously different soil ECs as shown in Figure 5d. In this case, poor comparability was found between the soil ECs measured using different water to soil ratios. Meanwhile, it did not represent the actual soil EC. By contrast, the new representation method of soil EC using EIS in this study denoted the real-time soil conductivity without any pretreatment. Though this study confirmed that the soil EC was representative of a certain soil type, some further verification needs to be done in other soil types. However, the new representation method put forward in this study represents a new methodology for the measurement of *in situ* soil/sediment EC. 

The environmental monitoring of polluted underground media is a focus issue for environmental geophysics. The conductivity of soils/sediments invaded by pollutants (e.g., oils or heavy metals) will change. Using the resistivity of polluted soil/sediment to probe the polluted zone is an important contamination survey approach [17]. Furthermore, the resistivity detected by the conventional method shows poor pragmaticality in contaminated site remediation and is time-consuming [18]. A new representation of soil resistance based on EIS in soil RMA was constructed from the viewpoint of the reasons mentioned above and is here named resistance measurement apparatus (RMA). The *in situ* soil resistivity as well as EC can be determined using RMA, which could indicate the soil EC for a variety of actual moisture contents. Moreover, this method was easy to carry out without complex pretreatments such as soil desiccation, mixture oscillation or centrifugation. In addition to soil, the *in situ* sediment EC also can be evaluated. For sediments, the EC measured using RMA could exhibit a greater advantage due to the use of actual water above the sediment. Additionally, the RMA also can be developed for remote and real-time sensing *in situ* monitoring. For example, 3~5 anodes were inserted into the sediment at different depths, while the air-cathode was placed in the superficial water. Each pair of electrodes (anode and cathode) were connected with the electrochemical workstation to measure the EIS at astated time. Then the sediment EC was obtained using the approach put forward in this study. It is well known that the Wenner-Schlumberger array is a good method for determining the EC of soils/sediments. RMA has some advantages in this study. For example, it is feasible to analyze the *in situ* and real-time change of EC in soil applications as sediment microbial remediating cells, or plant MFCs, and so on. Certainly, this new method needs to be further optimized in the future. Furthermore, the effect of insoluble conductive amendments (e.g., metal powders or carbon fibers) on the EC of soil or sediment can be investigated by using RMA rather than the common method (soil extraction). However, the RMA was only verified for a certain moisture content range from 9.1% to 37.5% (common water content of soil). Therefore, further verification need be done in subsequent tests. 

## Figures and Tables

**Table 1:** Characteristics of tested soil.

Index	Value
Soil density (g·cm^−3^)	1.47
Soil total porosity (%)	45%
pH	8.26
Conductivity (ms·cm^−1^)	1.99
Salt content (%)	2.86
Sand	30%
Coarse	23%
Fine	25%

**Table 2:** *R*_s_, *R*_ct_ and capacitance at different moisture contents of soil samples. *R*_ct_ is the sum of *R*_cathode_ and *R*_anode._ *C* is the sum of *C*_cathode_ and *C*_anode_.

MoistureContent	Distance from Air-Cathode	1 cm	2 cm	3 cm	4 cm	5 cm
9.1%	*R*_s_ (Ω)	34.82	58.23	29.02	61.04	69.77
	*R*_ct_ (Ω)	72.99	246.4	105.2	-	1229
	*C* (Ω^−1^·s^n^)	0.006409	0.000575	0.006406	0.00368	0.003364
16.7%	*R*_s_ (Ω)	23.37	33.81	39.92	41.5	44.16
	*R*_ct_ (Ω)	8.651	150.7	39.54	40.06	104.2
	*C* (Ω^−1^·s^n^)	0.001198	0.000211	0.000834	0.000616	0.003778
23.1%	*R*_s_ (Ω)	3.896	6.607	7.233	9.199	7.938
	*R*_ct_ (Ω)	6.378	189.9	9.005	67.74	7.238
	*C* (Ω^−1^·s^n^)	0.000188	0.000199	0.00066	0.000259	0.005059
28.6%	*R*_s_ (Ω)	3.388	5.582	6.853	8.762	8.656
	*R*_ct_ (Ω)	13.97	277	18.28	36.42	8.126
	*C*(Ω^−1^·s^n^)	0.000318	0.000204	0.000497	0.000273	0.003022
33.3%	*R*_s_ (Ω)	3.502	4.614	5.74	6.963	6.518
	*R*_ct_ (Ω)	55.93	602.2	55.72	57.71	8.201
	*C* (Ω^−1^∙s^n^)	0.000464	0.000211	0.000526	0.000369	0.004052
37.5%	*R*_s_ (Ω)	3.373	4.337	5.758	6.56	6.075
	*R*_ct_ (Ω)	55.03	498.1	56.89	48.99	7.79
	*C* (Ω^−1^·s^n^)	0.000457	0.000243	0.000737	0.000448	0.005716

**Table 3:** Conductivity and resistivity calculated from the linear fitting slope of *R*_s_ and distance from air-cathode at different moisture contents of soil samples.

Moisture Content (%)	Slope of Fitting	Resistivity (Ω·cm)	Conductivity (mS·cm^−1^)
9.1	5.189	407.543	2.454
16.7	5.295	415.868	2.405
23.1	1.666	130.847	7.642
28.6	1.667	130.926	7.638
33.3	1.126	88.436	11.308
37.5	1.029	80.817	12.374

**Table 4:** Conductivity and resistivity calculated from the linear fitting slope of *R*_s_ and distance from air-cathode in Figure 5.

Carbon Mesh Layer	Moisture Content (%)	Slope of Fitting	Resistivity (Ω·cm)	Conductivity (mS·cm^−1^)
Single	28.6	2.95308	231.934	4.312
Multiple	28.6	2.90248	227.960	4.387

## Supplementary Materials

The following are available online at www.mdpi.com/1424-8220/16/5/625/s1, Figure S1: Duplicate measurement of samples showed in Figure 2, Table S1: *R*_s_, *R*_ct_ and capacitance of duplicate measurements shown in Table 2.

## Abbreviations

The following abbreviations are used in this manuscript:

MFCs	Microbial fuel cells
EC	Electrical conductivity
EIS	Electrochemical impedance spectroscopy
RMA	Resistance measurement apparatus
*R*_s_	Ohmic resistance
*R*_ct_	Charge transfer resistance
C	Capacitance
CL	Catalyst layer
GDL	Gas diffusion layer
