# Supplementary material for: In Situ Representation of Soil/Sediment Conductivity Using Electrochemical Impedance Spectroscopy
Source: Sensors (Basel). 2016 Apr 30;16(5):625. doi: 10.3390/s16050625 (PMC4883316; doi:10.3390/s16050625)
Supplement: Supplementary file 1 [file sensors-16-00625-s001.pdf]

Supplementary Materials: *In Situ* Representation of Soil/Sediment Conductivity Using Electrochemical Impedance Spectroscopy

Xiaojing Li, Xin Wang, Qian Zhao, Yueyong Zhang and Qixing Zhou

Table S1 R_s, R_{ct} and capacitance of duplicate measurement showed in Table 2.

Moisture Content	Distance from Air-Cathode	1 cm	2 cm	3 cm	4 cm	5 cm
9.10%	**R_s (Ω)**	**41.25**	**62.58**	**44.2**	**65.46**	**66.94**
	R_{ct} (Ω)	80.59	113.7	14.45	2117	1066
	C ($\Omega^{-1}\cdot s^n$)	0.009935	0.006599	0.01182	0.00331	0.00327
16.70%	**R_s (Ω)**	**19.49**	**33.48**	**41.37**	**43.87**	**47.93**
	R_{ct} (Ω)	111.351	181.54	102.27	99.54	60.03
	C ($\Omega^{-1}\cdot s^n$)	0.060721	0.101225	0.061747	0.071812	0.070578
23.10%	**R_s (Ω)**	**3.654**	**4.329**	**7.122**	**8.554**	**9.351**
	R_{ct} (Ω)	56.587	171.1	27.4	112.19	4.35
	C($\Omega^{-1}\cdot s^n$)	0.064302	0.045649	0.217757	0.030561	0.205322
28.60%	**R_s (Ω)**	**3.265**	**4.527**	**6.736**	**8.667**	**8.899**
	R_{ct} (Ω)	64.47	254.8	49.17	59.31	7.155
	C ($\Omega^{-1}\cdot s^n$)	0.069352	0.041354	0.133952	0.1241	0.163727
33.30%	**R_s (Ω)**	**2.454**	**4.182**	**5.868**	**6.949**	**6.975**
	R_{ct} (Ω)	47.58	687.7	182.75	41.22	6.008
	C ($\Omega^{-1}\cdot s^n$)	0.071042	0.001387	0.046463	0.055404	0.176602
37.50%	**R_s (Ω)**	**2.364**	**3.738**	**4.889**	**6.448**	**6.641**
	R_{ct}(Ω)	46.9	495.397	82.43	40.02	4.877
	C ($\Omega^{-1}\cdot s^n$)	0.073304	0.000345	0.023344	0.068155	0.275701

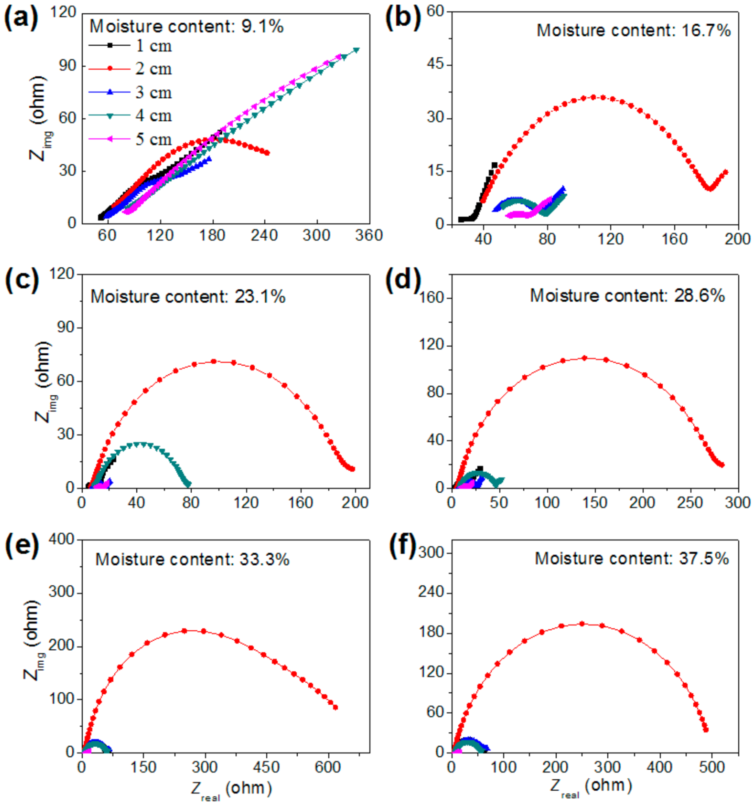

Figure S1. Duplicate measurements of samples shown in Figure 2. Nyquist plots (**a**–**f**) of soils of different moisture contents at open circuit potential and the equivalent circuit (**g**) for simulating electrochemical impedance spectroscopy.
